# Darolutamide-mediated phospholipid remodeling induces ferroptosis through the SREBP1-FASN axis in prostate cancer

**DOI:** 10.7150/ijbs.101039

**Published:** 2024-09-03

**Authors:** Bingheng Li, Bisheng Cheng, Hao Huang, Shanhe Huang, Shunli Yu, Zean Li, Shirong Peng, Tao Du, Ruihui Xie, Hai Huang

**Affiliations:** 1 Department of Urology, Sun Yat-sen Memorial Hospital, Sun Yat-sen University, Guangzhou 510120, China.; 2 Department of Urology, Nanfang Hospital, Southern Medical University, Guangzhou, China.; 3 Department of Obstetrics and Gynecology, Sun Yat-Sen Memorial Hospital, Sun Yat-Sen University, Guangzhou, 510120, Guangdong, China.; 4 Department of Urology, The Sixth Affiliated Hospital of Guangzhou Medical University, Qingyuan People's Hospital, Qingyuan,511518, Guangdong, China.

**Keywords:** darolutamide, phospholipid remodeling, ferroptosis, prostate cancer

## Abstract

Darolutamide, an androgen receptor inhibitor, has been approved by the Food and Drug Administration (FDA) for the treatment of prostate cancer (PCa), especially for patients with androgen receptor mutations. Owing to the unique lipidomic profile of PCa and the effect of darolutamide, the relationship between darolutamide and ferroptosis remains unclear. The present study showed that darolutamide significantly induces ferroptosis in AR^+^ PCa cells. Mechanistically, darolutamide promotes ferroptosis by downregulating SREBP1, which then inhibits the transcription of FASN. FASN knockdown modulates phospholipid remodeling by disrupting the balance between polyunsaturated fatty acids (PUFAs) and saturated fatty acids (SFAs), which induces ferroptosis. Clinically, SREBP1 and FASN are significantly overexpressed in PCa tissues and are related to poor prognosis. Moreover, the synergistic antitumor effect of combination therapy with darolutamide and ferroptosis inducers (FINs) was confirmed in PCa organoids and a mouse xenografts model. Overall, these findings revealed a novel mechanism of darolutamide mediated ferroptosis in PCa, laying the foundation for the combination of darolutamide and FINs as a new therapeutic strategy for PCa patients.

## Introduction

Prostate cancer (PCa) is one of the most common cancers in men and will remain the leading cause of death worldwide by 2024 1. While patients diagnosed with localized PCa usually have favorable survival times, the 5-year survival of metastatic patients is approximately 30% 2. Because the androgen receptor (AR) signaling pathway plays a vital role in PCa progression, androgen-deprivation therapy (ADT) is a standard treatment for patients with high-risk localized or advanced PCa. However, advanced patients ultimately progress to castration-resistant prostate cancer (CRPC), due to AR signaling reactivation or AR mutations. AR signaling-targeting agents (enzalutamide and abiraterone) have been approved by the FDA for the treatment of CRPC, but treatment failure inevitably occurs, resulting in the progression of patients to more aggressive subtypes 3, 4. In this scenario, a new second-generation antiandrogen inhibitor, darolutamide, has been used to treat nonmetastatic CRPC. Compared to other antiandrogens, darolutamide is a distinct androgen-receptor inhibitor that has lower blood-brain barrier penetration and side effects 5. More importantly, darolutamide has a unique and high affinity for AR splice variants, such as androgen receptor variant 7 (AR V7) or the AR F877L mutation 6. Recently, some reports have shown that ADT combined with ferroptosis inducers results in better tumor suppression 7, 8. However, the mechanism of this combination and relationship between darolutamide and ferroptosis is still unclear. Hence, the effect of the combination of darolutamide and ferroptosis inducers in specific PCa patients with AR mutations remains to be elucidated.

Ferroptosis is an iron-dependent programmed cell death, process characterized by the accumulation of lipid peroxides and excessive amounts of reactive oxygen species (ROS) 9. Additionally, in terms of cell morphology, biochemistry, and genetics, ferroptosis is different from necrosis, apoptosis, and autophagy due to its distinct properties. In contrast to a ruptured nucleus, the hallmarks of ferroptosis are a reduction in or loss of mitochondrial cristae, condensation of the mitochondrial membrane, and rupture of the mitochondrial membrane, as shown by morphologic observations via electron microscopy 10. The excess labile iron pool generates hydroxyl radicals through the Fenton reaction, resulting in the lethal accumulation of membrane lipid peroxidation and ROS. Although certain antiandrogens can result in mitochondrial dysfunction and induce ROS production 11, 12, elucidating the underlying association and mechanism between darolutamide and phospholipid metabolism is urgently needed.

Membrane lipid peroxidation plays vital roles in the process of ferroptosis. Polyunsaturated fatty acids (PUFAs), especially PUFA-phospholipids, are highly sensitive to peroxidation and are considered signals that initiate ferroptosis. In contrast, increased levels of monounsaturated fatty acids (MUFAs) and saturated fatty acids (SFAs) in the membrane protect cells from lipid oxidation and ferroptosis onset 13, 14. Hence, the enzymes and associated genes that regulate PUFAs or SFAs metabolism may modulate cellular vulnerability to ferroptosis. Sterol regulatory element binding protein 1 (SREBP1), a key regulator of lipid metabolism, and long chain acyl CoA synthetase 4 (ACSL4), which is a positive marker of ferroptosis, have been reported to play a role in antioxidant process 15, 16. Moreover, stearoyl-coenzyme A (CoA) desaturase-1 (SCD1), the regulator of unsaturated fatty acid metabolism, can synergize MUFAs, resulting in an anti-ferroptosis effect 17, 18. However, the crosstalk between darolutamide and the SFA/PUFA ratio in PCa remains unknown.

The present study demonstrated that darolutamide decreases SREBP1 expression to inhibit fatty acid synthase (FASN) transcription, which induces lipid peroxidation and ferroptosis onset, leading to suppression of PCa tumorigenesis. FASN knockdown increases the percentage of PC/PE-PUFAs, but decreases the percentage of PC/PE-MUFAs and PC/PE-SFAs, resulting in an imbalance between SFAs and PUFAs to induce ferroptosis. Moreover, the safety and efficacy of darolutamide combined with ferroptosis inducers (erastin and RSL-3) were evaluated *in vivo* and *in vitro*, and the results demonstrated that it has synergistic anti-tumorigenesis effects on PCa. In summary, the present study revealed the underlying mechanism by which darolutamide induces ferroptosis through the SREBP1-FASN axis in PCa, suggesting that the combination of darolutamide with FINs could be a promising therapeutic strategy for PCa patients with AR mutations.

## Materials and methods

### Cell lines and cell culture

The human C4-2 and LNCaP prostate cancer cell lines were purchased from American Type Culture Collection (ATCC, Manassas, VA). All cells were cultured in RPMI medium (Gibco, Shanghai, China) supplemented with 10% FBS and 10% penicillin/streptomycin (MCE, Shanghai, China) at 37 °C with 5% CO2.

### Chemicals

Darolutamide (S7559), necrostatin-1(S8037), erastin (S7242), RSL-3 (S8155) and ferrostatin-1 (S7243) were purchased from Selleck (Shanghai, China). Exogenous palmitic acid (HY-N0830-10) was purchased from MedChemExpress.

### Cell proliferation, and colony formation

For cell proliferation, cells were seeded in 96-well plates in triplicate and the Cell Counting Kit 8 (CCK-8) assay was performed according to the manufacturer's protocol. To elucidate the role of darolutamide in driving ferroptosis, 4000 cells were plated and treated with darolutamide and/or various cell death inhibitors (Z-VAD-FMK, necrostatin-1, and ferrostatin-1) for the indicated times. To assess the combination effect of darolutamide and FINs (erastin and RSL-3), 4000 cells were plated and treated with darolutamide, erastin, or RSL-3, as a single agent or in combination for 24 h. Combination indices (CIs) were analyzed by CompuSyn software using the Chou-Talalay method. For colony formation, 600 cells per well in 6-well plates were cultured and cells were treated with erastin (20 µM), RSL-3 (100 nM), darolutamide, or their combination. After 15 days, colonies were fixed with 95% methanol and stained with 0.1% crystal violet.

### Transmission electron microscopy (TEM)

Cells were cultured in 10 cm cell dishes, collected, pelleted, and fixed with 2.5% glutaraldehyde (Solaribio) for 2 h at 4 °C. TEM imaging was performed by Servicebio (Wuhan, China).

### Lipid peroxidation and reactive oxygen species detection

Lipid peroxidation was detected using a BODIPY 581/591 C11 kit (Thermo Fisher Scientific, Waltham, MA, USA). Cells were seeded in triplicate in 6-well plates for 24 h. Chemical compounds were then added for the indicated times. The treated cells were stained with 5 μM C11-BODIPY (581/ 591). The intensity of BODIPY 581/591 C11 was measured by flow cytometry at excitation/emission wavelengths of 488/510 (traditional FITC filter set) or by fluorescence microscope. A reactive oxygen species (ROS) assay kit (Beyotime Biotechnology) was used to detect the intracellular ROS levels according to the manufacturer's protocol. The relative lipid peroxidation and ROS levels were analyzed by FlowJo software.

### Malondialdehyde (MDA) assay

The relative MDA level was measured using an MDA activity assay kit (Beyotime Biotechnology, China) according to the manufacturer's protocol. Briefly, the indicated cells were lysed and centrifuge to collect the supernatant for detecting MDA levels. BCA protein assay kit (Beyotime Biotechnology, China) was used to detect the protein concentrations.

### RNA interference

Cells were transfected with siRNAs (GenePharma, Shanghai, China) specifically targeting AR, SREBP1, or FASN using Lipofectamine Rimax (Life Technologies, USA) according to the manufacturer's instructions. The sequences of the antisense siRNAs are shown in the Supplementary information.

### Cell transfection and lentiviral infection

For SREBP1 or FASN overexpression, the coding sequences of human SREBP1 or FASN were cloned and inserted into the lentiviral vector pCDH. Then, we generated these lentiviruses according to previous instructions in our laboratory 18. We generated shAR, shSREBP1 and shFASN knockdown plasmids with the Plko.1 vector. The shCtrl and shAR, shSREBP1 and shFASN targeting sequences are shown in the Supplementary information.

### RNA extraction, reverse transcription, and qRT‒PCR

Total RNA was isolated using an RNA-Quick Purification Kit (RN001, Shanghai, China) according to the manufacturer's instructions. cDNA was synthesized using HiScript III All-in-One RT SuperMix Perfect for qRT-PCR (Vazyme, Nanjing, China). The expression of mRNA was measured with an ABI QuantStudio Sequence Detection System (Applied Biosystems). The primers used to amplify the target genes are listed in the Supplementary Information.

### Western blotting analysis

Western blotting analysis was performed as previously described 19. Membranes were incubated overnight at 4 °C with primary antibodies against SREBP1 (1:1000, 14088-1-AP, Proteintech), AR (1:1000, 22089-1-AP, Proteintech), FASN (1:1000, 10624-2-AP, Proteintech) and GAPDH (1:1000, A19056, ABclonal Biotechnology). After incubation with an anti-rabbit secondary antibody (1:5000, AS014, ABclonal Biotechnology), the bands were visualized using a sensitive ECL kit (PK10002, Proteintech).

### Chromatin immunoprecipitation

Chromatin immunoprecipitation (ChIP) was performed using a ChIP A/G Assay Kit (P2080S, Beyotime Biotechnology), as previously described 20. Briefly, the transfected cells were fixed with 1% formaldehyde and subjected to ultrasonication. The indicated antibodies (anti-IgG, anti-SREBP1, or RNA polymerase II) were then added to the lysates. The chromatin fragments were harvested after washing with low salt, high salt, LiCl, and elution buffer. The primers used for ChIP-qPCR are listed in the Supplementary information.

### Lipid metabolite analysis

The relative lipid content was determined by Hangzhou RepuGene Co., Ltd. Briefly, the lipids content was determined by a Q Exactive Orbitrap mass spectrometer coupled to a Dionex UltiMate 3000 LC system (Thermo Scientific) and an ACQUITY UPLC CSH C18 (1.7 um, 2.1*100 mm) liquid chromatography column was used. On the basis of MS/MS data, lipid species were identified via “Lipid Search”.

### Prostate cancer organoid culture

Prostate organoid culture was performed as previously described 20. Cells were resuspended in complete culture media mixed with a 1:2 volume of Matrigel matrix (BD Biosciences, Cat# 356234). Complete medium was added after the Matrigel polymerized followed by culture at 37 °C.

### Xenograft mouse models

The animal experiments were approved by the Institutional Animal Care and Use Committee of Sun Yat-Sen University (approval no. AP20240080). Male 5- to 6-week-old BALB/c nude mice were purchased from the Charles River (Beijing, China). The tumor xenograft model procedure was performed as previously described 21. When the average tumor volume reached approximately 100 mm^3^, the mice were intraperitoneally injected with darolutamide (20 mg/kg, qd), RSL-3 (10 mg/kg, qd), or vehicle. This procedure was continued for 14 days. Mice were monitored daily, and the tumor volume was calculated using the following equation: volume = length × width^2^ × 1/2.

### Clinical samples

All PCa tumors and adjacent tissues were obtained from patients who underwent radical prostatectomy at Sun Yat-Sen Memorial Hospital (SYSMH) of Sun Yat-Sen University. The histologic and pathologic types of each sample were verified by two pathologists. All patients provided written informed consent, and the studies were approved by the Ethics Committees of Sun Yat-Sen Memorial Hospital of Sun Yat-Sen University (SYSEC-KY-KS-2020-201).

### Tissue microarray (TMA) and immunohistochemical (IHC) staining

TMAs for SREBP1, FASN, and 4-HNE were purchased from Zhuo Li Biotechnology Co., Ltd (Shanghai, China). The TMAs were used to analyze 80 PCa tissues and adjacent normal tissues. IHC staining was performed using a previously described standard method 22. Anti-SREBP1 (1:100,14088-1-AP, Proteintech), anti-FASN (1:100, 10624-2-AP, Proteintech), anti-4HNE (1:100, bs-6313R, Bioss), and anti-KI67 (1:200, 28074-1-AP, Proteintech) antibodies were used to measure gene expression. The H-score was calculated by the combining the percentage and intensity of cells according to previous protocols 22.

### Bioinformatics analysis

The GSE114016 and GSE148400 datasets are available in the Gene Expression Omnibus (GEO; http://www.ncbi.nlm.nih.gov/geo). Gene set enrichment analysis (GSEA) was performed with the clusterProfiler package in R. DEGs were considered to have a *P* value < 0.05 and log2 |FC| > 1. The indicated GO gene sets in the ranked gene lists represented by the normalized enrichment score (NES).

### Statistical analyses

The data from three independent experiments are presented as the means ± SD. GraphPad Prism (GraphPad Software, San Diego, CA, USA) was used to analyze the data. Two-tailed t tests or one-way ANOVA were performed to assess the group differences. *P* < 0.05 was considered to indicate statistical significance. (* *P* < 0.05, ** *P* < 0.01, and *** *P* < 0.001).

## Results

### Darolutamide promotes ferroptosis in AR^+^ PCa cells

**Figure 1:**
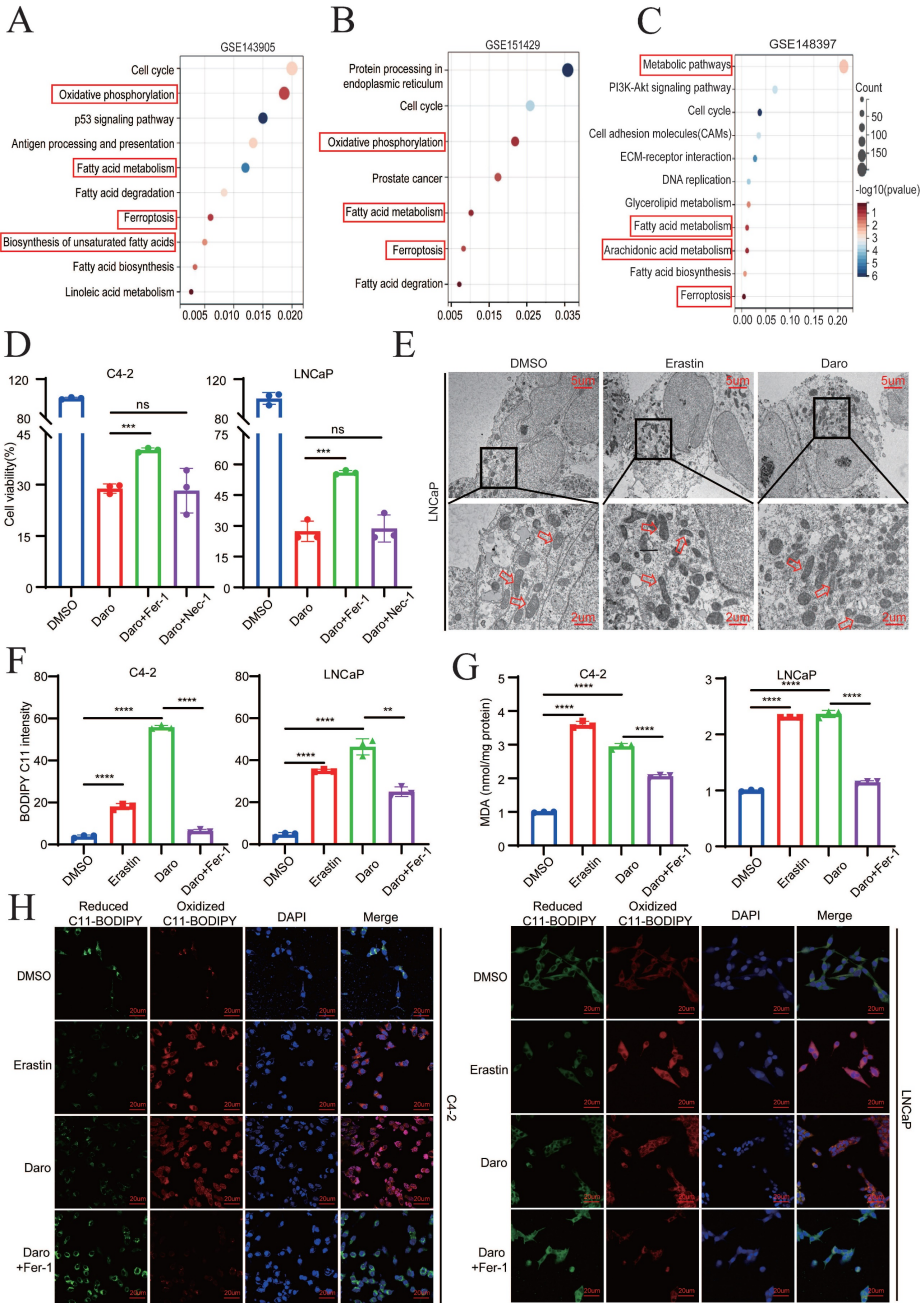
** Darolutamide promotes ferroptosis in AR+ PCa cells. A-B** KEGG pathway enrichment analysis of the DEGs between the AR WT and AR V-7 groups. **C** KEGG pathway enrichment analysis of the DEGs in the darolutamide-treated group. **D** Viability analysis of the indicated cells treated with darolutamide (C4-2,50 µM; LNCaP, 10 µM) plus cell death inhibitors Fer-1 (2µM) and Nec-1 (10µM) for 24h. **E** Representative images of mitochondria by TEM using LNCaP cells treated with erastin (20µM) or darolutamide (10µM). Scale bars represent 5 µm and 2 µm. **F** Levels of lipid peroxidation in the indicated groups examined using BODIPY C11 staining. **G** Analysis of MDA level in the indicated groups. Erastin treatment was used as a positive control. **H** Representative images of reduced and oxidized lipid in the indicated groups treated with BODIPY C11 staining. *p < 0.05, **p < 0.01, and ***p < 0.001.

Because most PCa patients eventually progress to CRPC, second-generation AR antagonists, such as darolutamide, have been approved by the FDA for CRPC treatment 5, 23. Given that darolutamide has greater affinity for AR mutations, the difference between the AR mutant and the AR WT was first analyzed. KEGG analysis demonstrated that ferroptosis and oxidative phosphorylation were significantly enriched, indicating that ferroptosis may play a critical role in PCa progression (**Fig. 1A-B**). The function of darolutamide was then determined in GSE148397 datasets using GEO2R. In total, 3200 DEGs were detected, including 1456 down-regulated genes and 1744 up-regulated genes (|logFC| > 1, P<0.05) (**Fig. S1A**). KEGG analysis indicated that genes related to arachidonic acid metabolism, fatty acid metabolism and ferroptosis were significantly enriched (**Fig. 1C**). Considering that arachidonic acid metabolism is also associated with ferroptosis, darolutamide may be involved in ferroptosis regulation. First, half-maximal darolutamide inhibitory concentration (IC50) was detected in C4-2 and LNCaP cells (**Fig. S1B**). To further verify whether darolutamide participates in ferroptosis, the cells were treated with darolutamide alone or in combination with a ferroptosis inhibitor ferrostain-1 (2 µM Fer‑1) or a necroptosis inhibitor necrostatin-1 (10 µM Nec-1). Compared to darolutamide treatment, Fer-1 clearly restored cell viability in C4-2 and LNCaP cells (**Fig. 1D**), indicating that darolutamide specifically modulates ferroptosis. Consistently, TEM analysis showed that darolutamide led to mitochondria shrinkage and the reduced mitochondrial cristae in LNCaP cells, which indicated that darolutamide induced ferroptosis in PCa (**Fig. 1E**). Moreover, darolutamide promoted the accumulation of total ROS and lipid peroxidation, which were reversed by a ferroptosis inhibitor (Fer-1) (**Fig. 1F, Fig. S1C**).

Additionally, darolutamide did not increase lipid peroxidation in AR^-^ PCa cells, which indicated its specific function in AR^+^ PCa cells (**Fig. S1D**). As the product of lipid peroxidation, MDA plays key roles in maintaining the balance of oxidation and reduction. As expected, darolutamide increased the MDA level, and Fer-1 blocked this effect, which further suggested that darolutamide promoted ferroptosis (**Fig. 1G**). As shown in **Figure 1H**, confocal imaging also revealed that darolutamide significantly increased oxidized lipid and decreased reduced lipid, which was rescued by Fer-1. Taken together, these findings indicated that darolutamide promotes ferroptosis in AR^+^ PCa cells.

### AR deletion sensitizes PCa cells to ferroptosis

**Figure 2:**
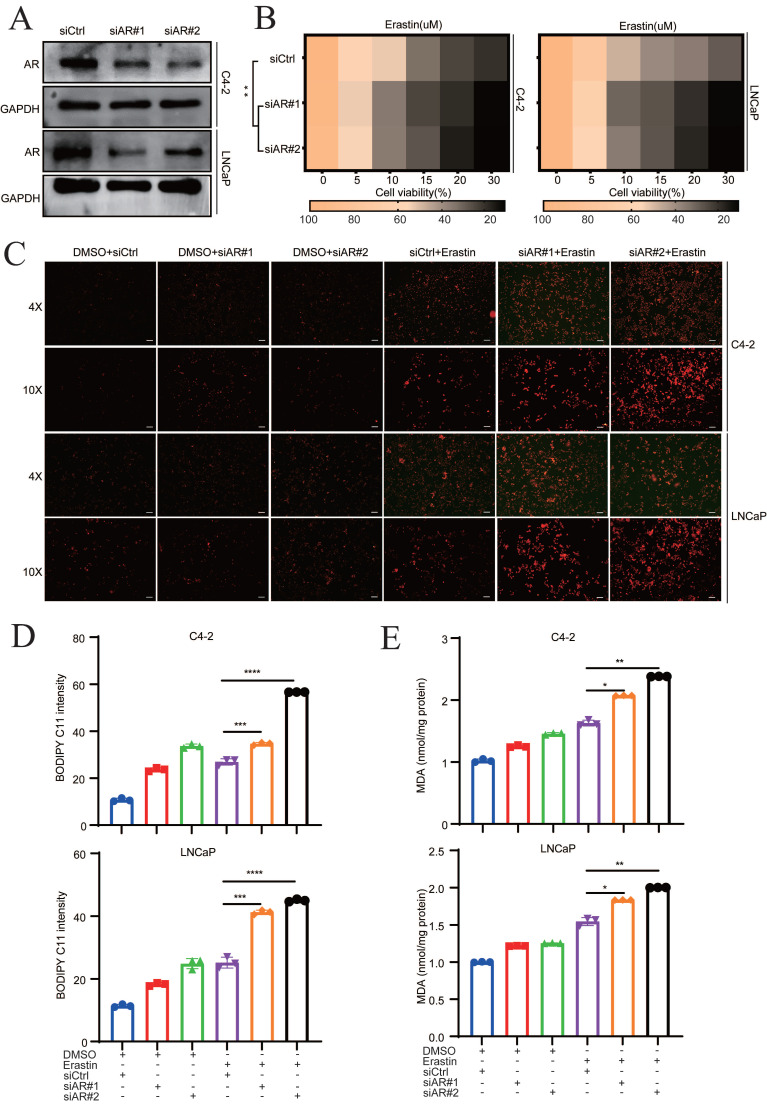
**AR deletion sensitizes PCa cells to ferroptosis. A** Immunoblot analysis of AR in the indicated cells treated with siCtrl and siRNA targeting AR. **B** Viability analysis of siAR cells treated with different concentrations of erastin (0, 5, 10, 15, 20, and 30 µM) for 24 h. **C** Representative images of cellular reactive oxygen species (ROS) in the indicated cells treated DCFH-DA probe. **D-E** Levels of BODIPY C11 (**D**), as measure of the lipid peroxidation, and MDA concentrations (**E**), examined using MDA assay kit in the indicated cells treated with or without erastin for 24h. *p < 0.05, **p < 0.01, and ***p < 0.001.

The mechanism of darolutamide is mainly derived from the inhibition of AR signaling pathways, which suppresses PCa progression 3, 5, 23. To investigate whether AR play a vital role in ferroptosis, siRNA was used to knockdown AR in C4-2 and LNCaP cells. The knockdown efficacy was confirmed by Western blot and qRT-PCR assays (**Fig. 2A, Fig. S2A**). AR knockdown in C4-2 and LNCaP cells significantly enhanced the effect of erastin, a ferroptosis inducer (**Fig. 2B**). Furthermore, fluorescence analysis showed that lipid peroxidation was markedly increased in the combination of AR knockdown cells with erastin treatment, compared with erastin group (**Fig. 2C**). Measurement of the lipid peroxidation and MDA levels demonstrated that AR knockdown significantly increased the sensitivity of both PCa cell lines to erastin (**Fig. 2D-E**). Collectively, these results indicated that darolutamide-mediated AR inhibition sensitizes PCa cells to ferroptosis.

### Darolutamide promotes ferroptosis by inhibiting SREBP1 expression

**Figure 3:**
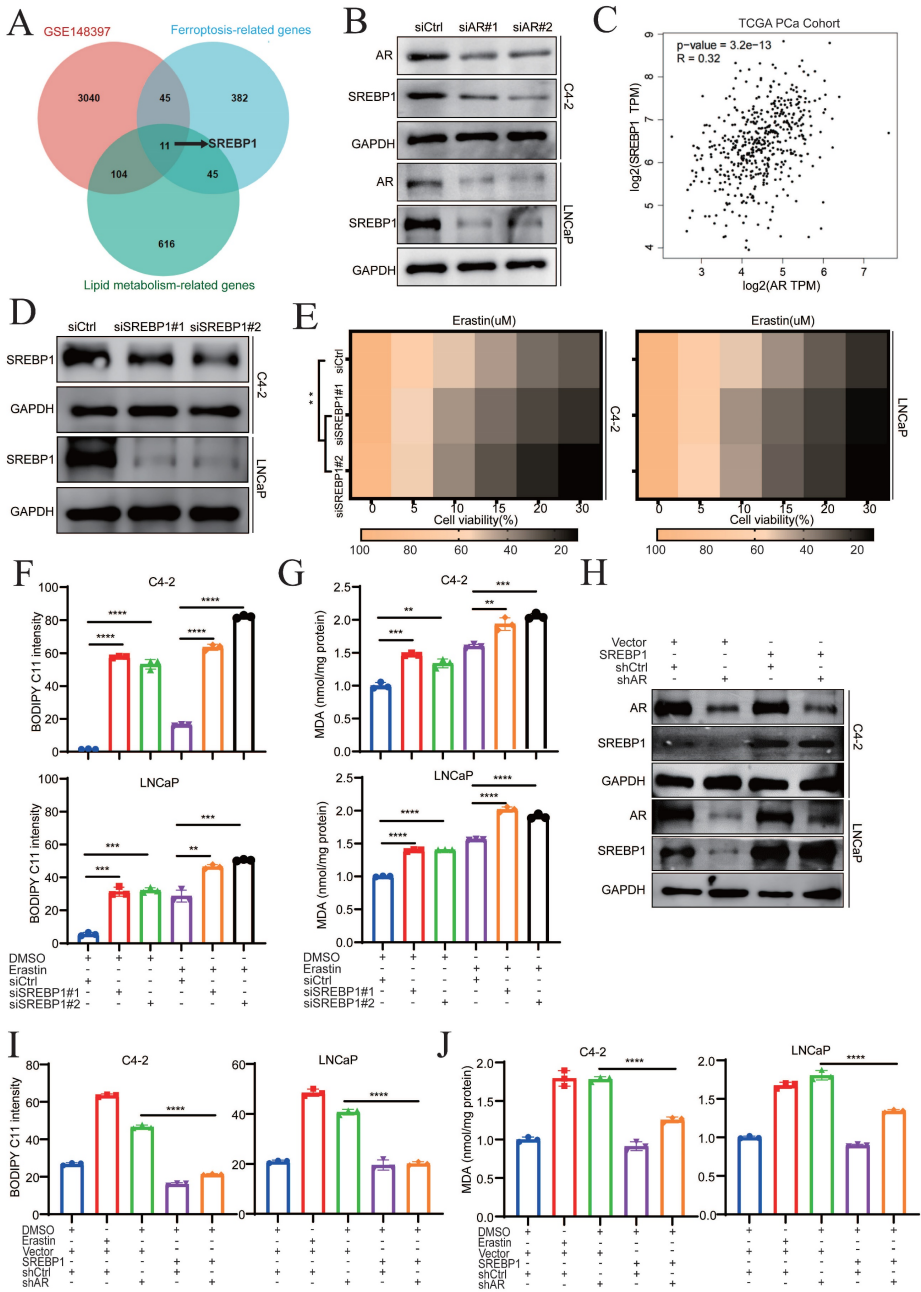
** Darolutamide promotes ferroptosis by inhibiting SREBP1 expression. A** Venn diagram showing the common candidate genes that overlapped with the GSE148397, ferroptosis-related genes, and lipid metabolism-related genes. **B** Immunoblot analysis of SREBP1 expression in AR-knockdown cells. **C** Analysis of the correlation between AR and SREBP1 in TCGA database. **D** Immunoblot analysis of SREBP1 expression in SREBP1 knockdown indicated cells. **E** Viability analysis of siSREBP1 indicated cells treated with different concentrations of erastin (0, 5, 10, 15, 20, and 30 µM) for 24 h. **F-G** Lipid peroxidation levels (**F**) and MDA concentrations (**G**) examined in the indicated cells treated with or without erastin for 24h. **H** Immunoblot analysis of AR and SREBP1 expression in the indicated cells; GAPDH served as a loading control. **I-J** Lipid peroxidation levels (**I**) and MDA concentrations (**J**) examined in the indicated cells by BODIPY C11 staining and MDA assay kit. *p < 0.05, **p < 0.01, and ***p < 0.001.

To explore the underlying mechanisms by which darolutamide promotes ferroptosis, potential fatty acid-related genes that can mediate ferroptosis-related biological processes were evaluated. First, three databases, namely, the GSE148397 datasets, ferroptosis-related gene database, and lipid metabolism-related gene database, were analyzed (**Supplementary Data 1**), using venn diagram, which identified 11 genes (**Fig. 3A**). Among those genes, only SREBP1 was positively related to the Gleason score and was highly expressed in PCa tissues (**Fig. S3A-C**). Because SREBP1 regulates ferroptosis and lipid peroxidation 16, darolutamide may induce ferroptosis by modulating SREBP1. To verify this hypothesis, AR was knocked down, which reduced the mRNA and protein levels of SREBP1 in C4-2 and LNCaP cells (**Fig. 3B, Fig. S3D**). Correspondingly, analysis of data from The Cancer Genome Atlas (TCGA) database revealed that AR expression was positively correlated with SREBP1 expression in PCa (**Fig. 3C**). Next, the relationship between SREBP1 and ferroptosis was explored in PCa cells. The efficiency of SREBP1 knockdown was measured by Western blot and qRT-PCR assays (**Fig. 3D, Fig. S3E**). SREBP1 knockdown combined with erastin treatment significantly induced cell death and increased lipid peroxidation and MDA levels, which demonstrated that SREBP1 mediated ferroptosis sensitivity in PCa cells (**Fig. 3E-G**). To further confirm that AR knockdown promotes ferroptosis via SREBP1, a pCMV-SREBP1 plasmid was constructed to overexpress SREBP1 in AR knockdown cells (**Fig. 3H**). Overexpression of SREBP1 significantly reversed the increases in lipid peroxidation and MDA levels caused by AR deficiency (**Fig. 3I-J**). These data indicated that darolutamide promotes ferroptosis by decreasing SREBP1 expression in PCa.

### Darolutamide facilitates ferroptosis through regulating SREBP1-FASN axis in PCa

**Figure 4:**
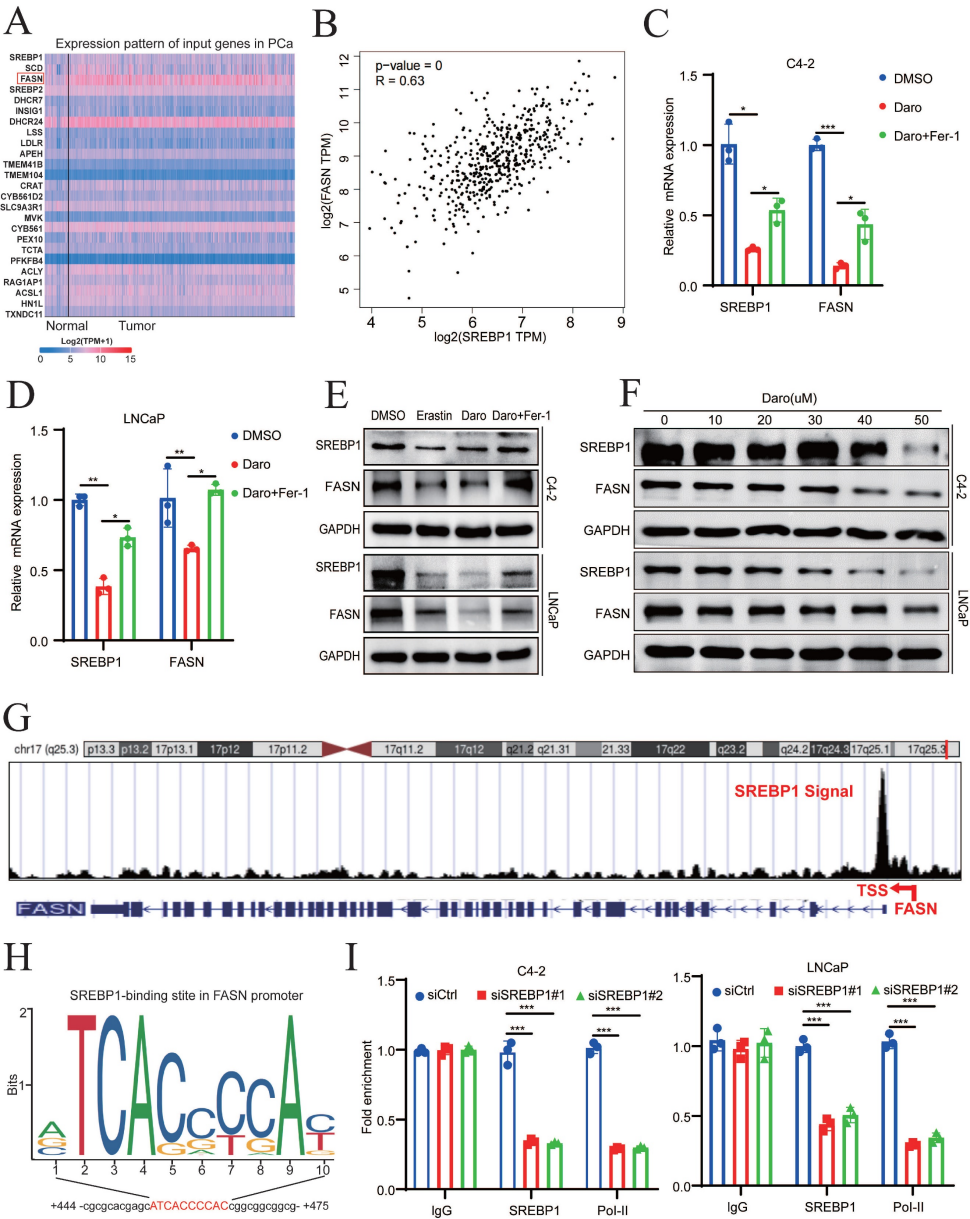
** Darolutamide facilitates ferroptosis by regulating the SREBP1-FASN axis in PCa. A** Top 25 genes related to SREBP1 expression in PCa in TCGA database. **B** Analysis of correlation between SREBP1 and FASN in TCGA database. **C-D** Relative mRNA level of SREBP1 and FASN in indicated cells treated with darolutamide (C4-2,50 µM; LNCaP, 10 µM) or darolutamide combined with Fer-1. **E** Immunoblot of SREBP1 and FASN expression in the indicated groups treated with darolutamide with or without Fer-1 for 24h. **F** Immunoblot of SREBP1 and FASN in C4-2 and LNCaP cells treated with different concentrations of darolutamide (0, 10, 20, 30, 40 and 50 µM) for 24h.** G** The UCSC genome bioinformatics site showed enrichment of SREBP1 in the promoter of FASN. **H** JASPAR predicted SREBP1-binding elements at the promoters of FASN. **I** ChIP-qPCR analysis of SREBP1 and RNA polymerase II (Pol-II) genomic occupancy at the FASN promoter after SREBP1 knockdown. *p < 0.05, **p < 0.01, and ***p < 0.001.

Because SREBP1 is a transcription factor, top 25 genes related to SREBP1 expression in PCa were identified using GEPIA 2.0 to elucidate the mechanism of SREBP1- mediated ferroptosis (**Fig. 4A**). Lipid synthesis-related genes, such as ACLY, ACC, FASN, and SCD1, were strongly positively correlated with SREBP1 expression in TCGA database (**Fig. 4B, Fig. S4A-C**). To explore whether these genes are regulated by AR, the inhibitory effect of darolutamide was first examined. Darolutamide significantly decreased the expression of AR downstream target genes, such as PSA and FKBP51, which were rescued by Fer-1 (**Fig. S4D-E**). In addition, the expression of SREBP1 was significantly lower in the darolutamide treatment groups than in the control group, and this decrease was reversed by ferroptosis inhibitors. Compared to other target genes, qRT-PCR analysis results showed that darolutamide significantly decreased the mRNA levels of FASN, and ferrostatin-1 rescued FASN expression (**Fig. 4C-D, Fig. S4F-G**). Similar trends were confirmed by Western blot analysis (**Fig. 4E**). As shown in **Figure. 4F**, darolutamide inhibited SREBP1 and FASN expression in a dose-dependent manner, further confirming their regulatory relationship. Because data analysis revealed an SREBP1 binding signal in the promoter region of FASN (**Fig. 4G**), JASPAR prediction was conducted, which identified the binding site of SREBP1 at the FASN promoter (**Fig. 4H**). ChIP assays further verified the direct binding of SREBP1 to the FASN promoter and the recruitment of RNA polymerase II (**Fig. 4I**). In addition, the knockdown of AR decreased the expression of both SREBP1 and FASN (**Fig. S4H-I**). Taken together, these results demonstrated that SREBP1 regulates FASN transcription in PCa.

**Figure 5:**
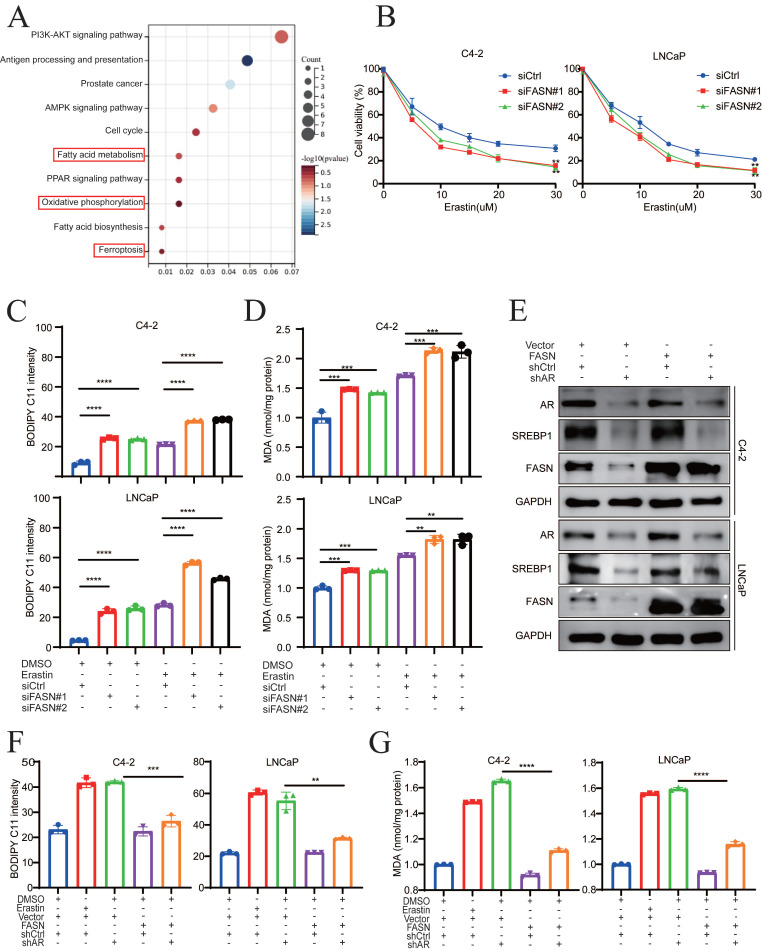
** FASN inhibition induces lipid peroxidation in PCa cells. A** KEGG enrichment analysis showed the pathways significantly enriched after treatment with FASN inhibition. **B** Viability analysis of FASN knockdown cells treated with different concentrations of erastin (0, 5, 10, 15, 20, and 30 µM) for 24 h. **C-D** Lipid peroxidation levels (**C**) and MDA concentrations (**D**) examined in the indicated cells treated with or without erastin for 24h.** E** Immunoblot analysis of SREBP1 and FASN expression in the indicated cells. **F-G** Lipid peroxidation levels (**F**) and MDA concentrations (**G**) examined in the indicated groups using flow cytometry and MDA assay kit. *p < 0.05, **p < 0.01, and ***p < 0.001.

Fatty acid synthase (FASN) is a key enzyme for the synthesis of fatty acids, and it is upregulated in various malignant cancers 6, 24-26. Lipidomic analysis revealed that FASN inhibition is associated with the accumulation of polyunsaturated fatty acids (PUFAs) in phosphatidylcholines (PCs) and lysophosphatidylcholine (lyso-PCs) 26. To obtain mechanistic insights into the relationship between FASN and ferroptosis in PCa, pathway enrichment was analyzed in AR+ PCa cells following FASN inhibition. KEGG revealed that fatty acid metabolism, oxidative phosphorylation and ferroptosis were significantly involved (**Fig. 5A**). Because oxidative phosphorylation is closely related to ROS and ferroptosis 27-29, FASN may play an important role in ferroptosis. Therefore, FASN was knocked down in C4-2 and LNCaP cells, and the efficacy of FASN knockdown was verified at both the mRNA and protein levels (**Fig. S5A-B**). Compared to that in the control group, FASN knockdown notably enhanced erastin treatment sensitivity (**Fig. 5B**). Similarly, FASN knockdown combined with erastin treatment effectively enhanced lipid peroxidation and MDA levels compared to the control group (**Fig. 5C-D, Fig. S5C**), which indicated that FASN knockdown potentiated the cytotoxic effect via ferroptosis. To elucidate the function of FASN-mediated ferroptosis in PCa, rescue experiments were performed in AR-deficient cells with ectopic overexpression of FASN (**Fig. 5E**). Lipid peroxidation and MDA levels were significantly suppressed by FASN overexpression in the AR knockdown group (**Fig. 5F-G**). Collectively, these findings indicated that darolutamide promotes ferroptosis by regulating the SREBP1-FASN axis in PCa.

### FASN inhibition mediates the SFA/PUFA ratio to regulate ferroptosis in PCa

**Figure 6:**
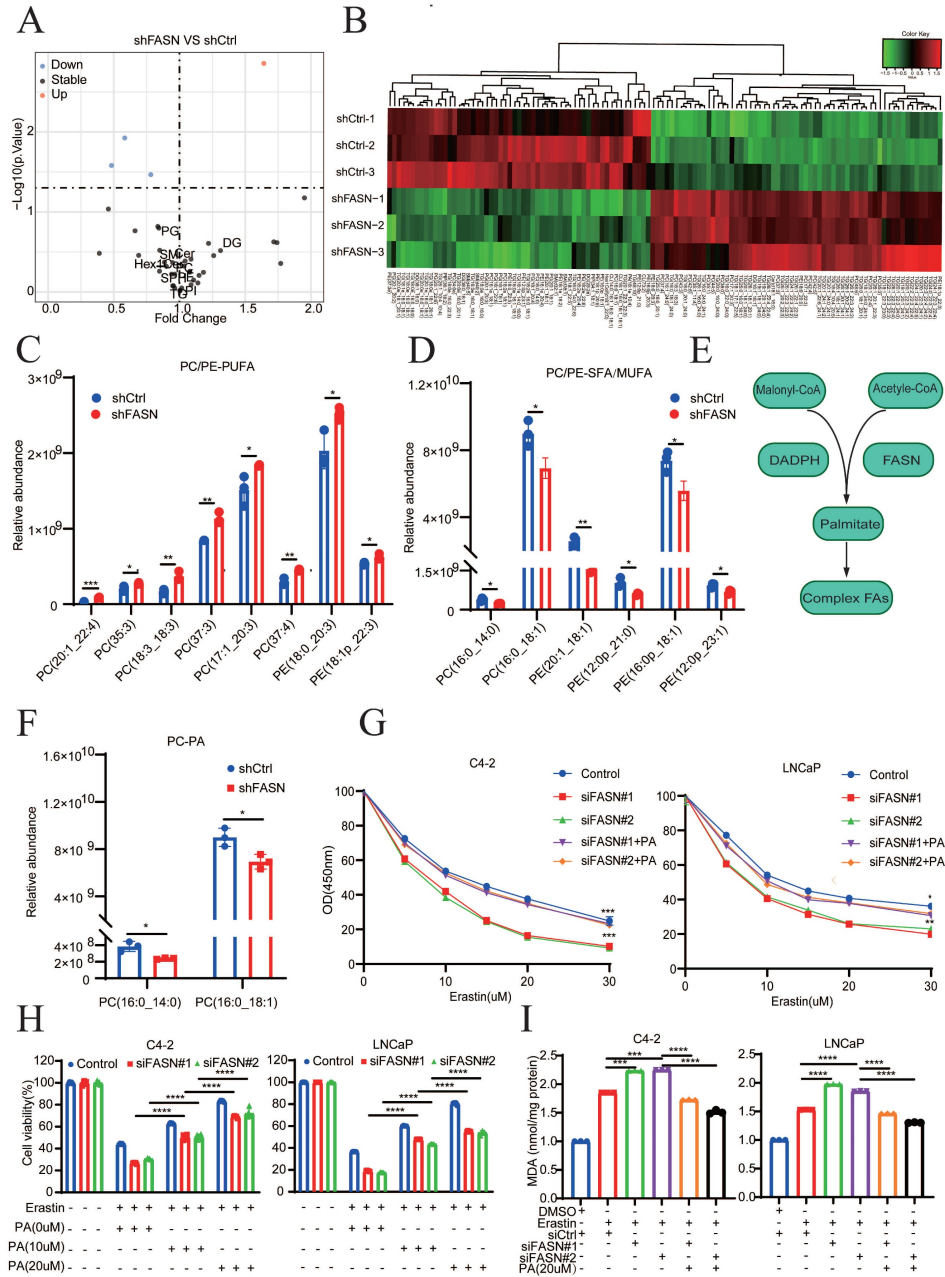
** FASN inhibition mediates the SFA/PUFA ratio to regulate ferroptosis in PCa. A** Lipidomic analysis of lipid classes by MS/MS in shCtrl and shFASN LNCaP cells. **B** Heatmap analysis, using liquid chromatography‒mass spectrometry (LC‒MS), of membrane phospholipids with different FAs analysis. **C-D** Quantification of the most abundant PE/PC-MUFAs and PE/PC-PUFAs in shCtrl and shFASN cells. **E** Schematic of the synthesis of palmitate (FA 16:0). **F** Quantification of PC-PA in shCtrl and shFASN cells.** G** Viability analysis of siFASN C4-2 and LNCaP cells, as indicated, which were treated with different concentrations of erastin and/or PA (20 µM) for 24 h. **H** Viability analysis of siCtrl and siFASN cells treated with or without erastin and different concentrations of PA (0, 10, and 20 µM) for 24 h. **I** MDA levels examined in the indicated groups. *p < 0.05, **p < 0.01, and ***p < 0.001.

FASN selectively regulates important metabolic genes, such as ACSL3, PLA2G4C, and LPCAT3, which remodel phospholipid metabolism and influence ferroptosis 30. To examine whether FASN regulates ferroptosis by mediating phospholipid metabolism in AR^+^ PCa cells, lipidomic analysis was performed. Most lipid compositions were not affected following FASN knockdown, which suggest that FASN did not change the total amount of lipid classes (**Fig. 6A**). However, FASN silencing selectively increased the accumulation of polyunsaturated FA-containing phosphatidylcholines (PC-PUFAs) and polyunsaturated FA-containing phosphatidylethanolamines (PE-PUFAs), while the levels of saturated FA-containing phosphatidylcholines (PC-SFAs) and saturated FA-containing phosphatidylethanolamines (PE-SFAs) were significantly decreased by FASN knockdown (**Fig. 6B-D**). Also, the level of monounsaturated FA-containing phosphatidylcholines (PC-MUFAs) and monounsaturated FA-containing phosphatidylethanolamines (PE-MUFAs) were decreased after silencing FASN expression. These results showed that FASN inhibition increases PUFA incorporation into PC or PE, which competitively decreases the PE/PC-MUFA and PC/PE-SFA contents, ultimately leading to a ferroptosis-sensitive cell state. Additionally, malonyl-CoA and the central carbon metabolite acetyl-CoA synthesized palmitic acid under the catalysis of FASN and the cofactor NADPH (**Fig. 6E**). Thus, the present study investigated whether exogenous fatty acids (FAs) influence ferroptosis sensitivity. Because palmitic acid (PA, FA 16:0) is the most abundant saturated fatty acid in humans and can be synthesized endogenously or provided by an exogenous diet 31, the changes in PA caused by FASN silencing were examined via lipidomic analyses. PA was significantly lower in the shFASN group, than in the control group (**Fig. S6A**). In addition, the amount of PC-PA, which is the major PL-SFA that is resistant to lipid peroxidation during ferroptosis, was significantly decreased in the FASN knockdown group (**Fig. 6F**). Oleic acid (OA FA 18:1) is also the most abundant cellular MUFA and can suppress lipid peroxidation 31. Compared to the control group, PC/PE-OA were significantly decreased in the shFASN group (**Fig. S6B**). These results suggested that FASN inhibition decreases MUFAs and SFA, and increases PUFA abundance to modulate ferroptosis in PCa. To further validate this hypothesis, the product of FASN (exogenous palmitate) was used to rescue the effect of FASN knockdown on ferroptosis. CCK-8 assays revealed that cell viability was significantly rescued by exogenous palmitate (PA) in a dose-dependent manner (**Fig. 6G-H**). Moreover, exogenous PA significantly reversed the level of lipid peroxidation induced by FASN knockdown in C4-2 and LNCaP cells (**Fig. 6I**). These data suggested that FASN inhibition not only increases the amount of PUFAs in PC or PE, but also decreases the incorporation of MUFAs or SFAs, including OA and PA, in PC and PE. In summary, FASN silencing regulates ferroptosis sensitivity by mediating the PUFA/SFA ratio in PCa.

### SREBP1 and FASN are elevated in PCa and associated with poor prognosis

**Figure 7:**
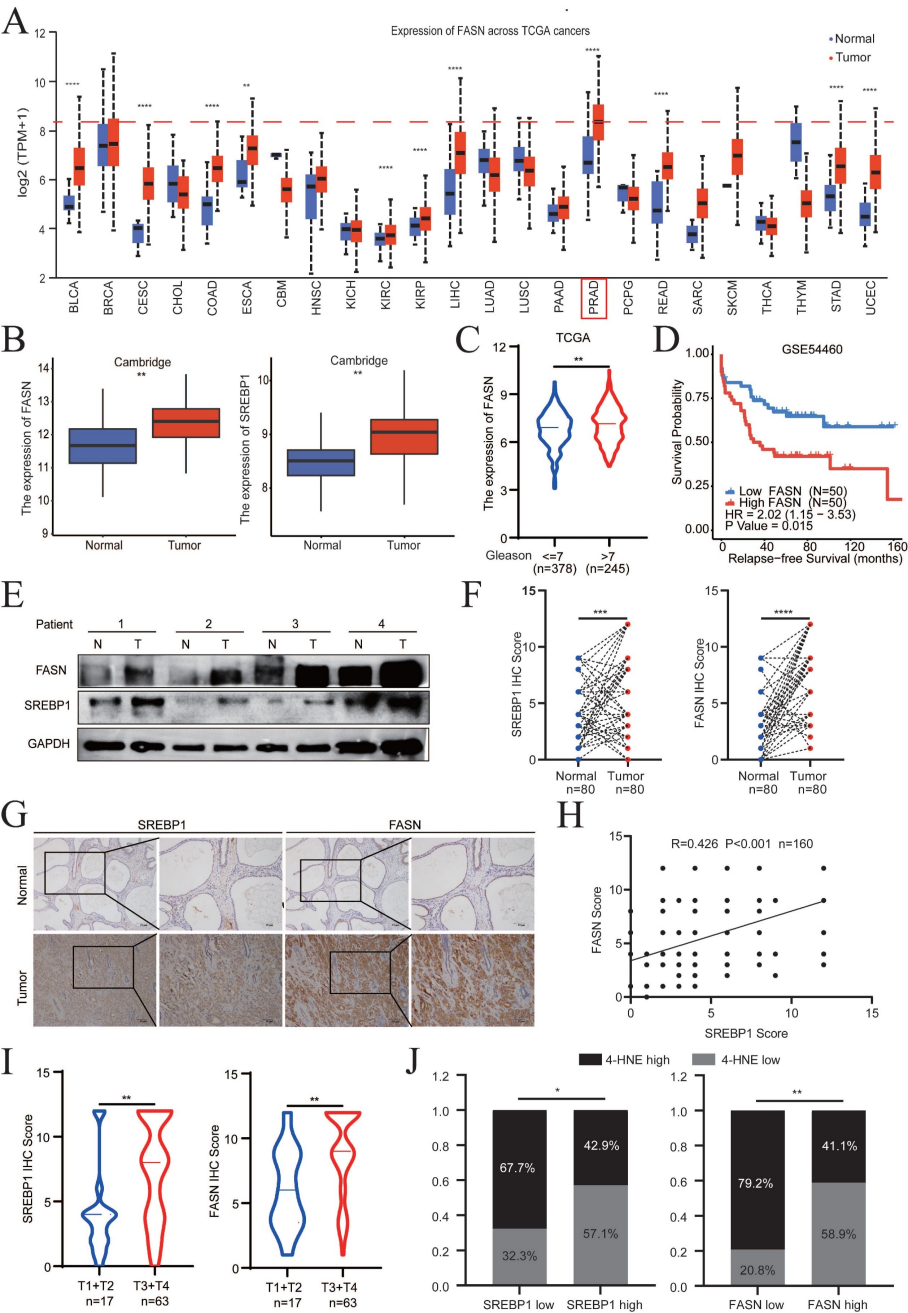
** SREBP1 and FASN are elevated in PCa and are associated with poor prognosis. A** Analysis of FASN expression in pan-cancer using The Cancer Genome Atlas datasets. **B** Comparison of SREBP1 and FASN expression between normal tissues and PCa tumors in the Cambridge database. **C** Analysis of FASN expression in different groups stratified according to Gleason score. **D** Kaplan‒Meier curves for the RFS of PCa patients with high versus low FASN expression in the GSE54460 datasets. **E** Immunoblot analysis of SREBP1 and FASN expression between normal tissues and PCa tumors in the SYSMH cohort.** F-G** Quantitative analysis and representative images of SREBP1 and FASN expression between adjacent tissues and PCa tumors in tissue microarrays.** H** Correlations analysis between SREBP1 and FASN expression using the Pearson correlation test. **I** Analysis of SREBP1 and FASN expression in the T1+T2 and T3+T4 groups.** J** Correlations analysis between SREBP1 and 4-HNE expression, as well as between FASN and 4-HNE expression, using Fisher's exact test. *p < 0.05, **p < 0.01, and ***p < 0.001.

Analysis of different cancer types revealed that FASN was significantly overexpressed in 11 of 24 tumor types (45.8%). Notably, PCa was the most common (ranked first) FASN-overexpressing tumor among all tumor types in TCGA database, suggesting the specific and important physiological functions of FASN in PCa (**Fig. 7A**). In addition, SREBP1 expression in PCa was relatively higher than that in other types of cancer (**Fig. S7A**).

Evaluation of the expression levels of SREBP1 and FASN in the Cambridge cohorts demonstrated that both SREBP1 and FASN were significantly upregulated in PCa tissues compared to normal tissues (**Fig. 7B**). In addition, the clinical significance and relapse-free survival (RFS) of FASN in PCa were further examined (TCGA database and GSE54460). The FASN expression level in PCa was positively associated with a higher Gleason score and poor DFS (**Fig. 7C-D**). Consistently, the above results were confirmed by clinical samples from PCa patients collected at Sun Yat-Sen Memorial Hospital (**Fig. 7E, Fig. S7B**). SREBP1, FASN, and 4-HNE expression was detected by IHC on PCa tissue microarrays (TMA) slides, which included 80 PCa tumor tissues and paired adjacent normal tissues. SREBP1 and FASN expression was significantly higher in PCa tissues than in adjacent normal tissues (**Fig. 7F-G**). Consistently, SREBP1 expression was positively correlated with FASN expression at the protein level (**Fig. 7H**). Moreover, SREBP1 and FASN expression was significantly higher in the T3+T4 group than in the T1+T2 group in PCa patients (**Fig. 7I**). In addition, the expression of SREBP1 or FASN was negatively correlated with that of 4-HNE (**Fig. 7J**). Taken together, these data demonstrated that SREBP1 and FASN are elevated in PCa and are associated with poor prognosis in PCa patients.

### Darolutamide and FINs synergistically sensitize PCa cells to cell death

**Figure 8:**
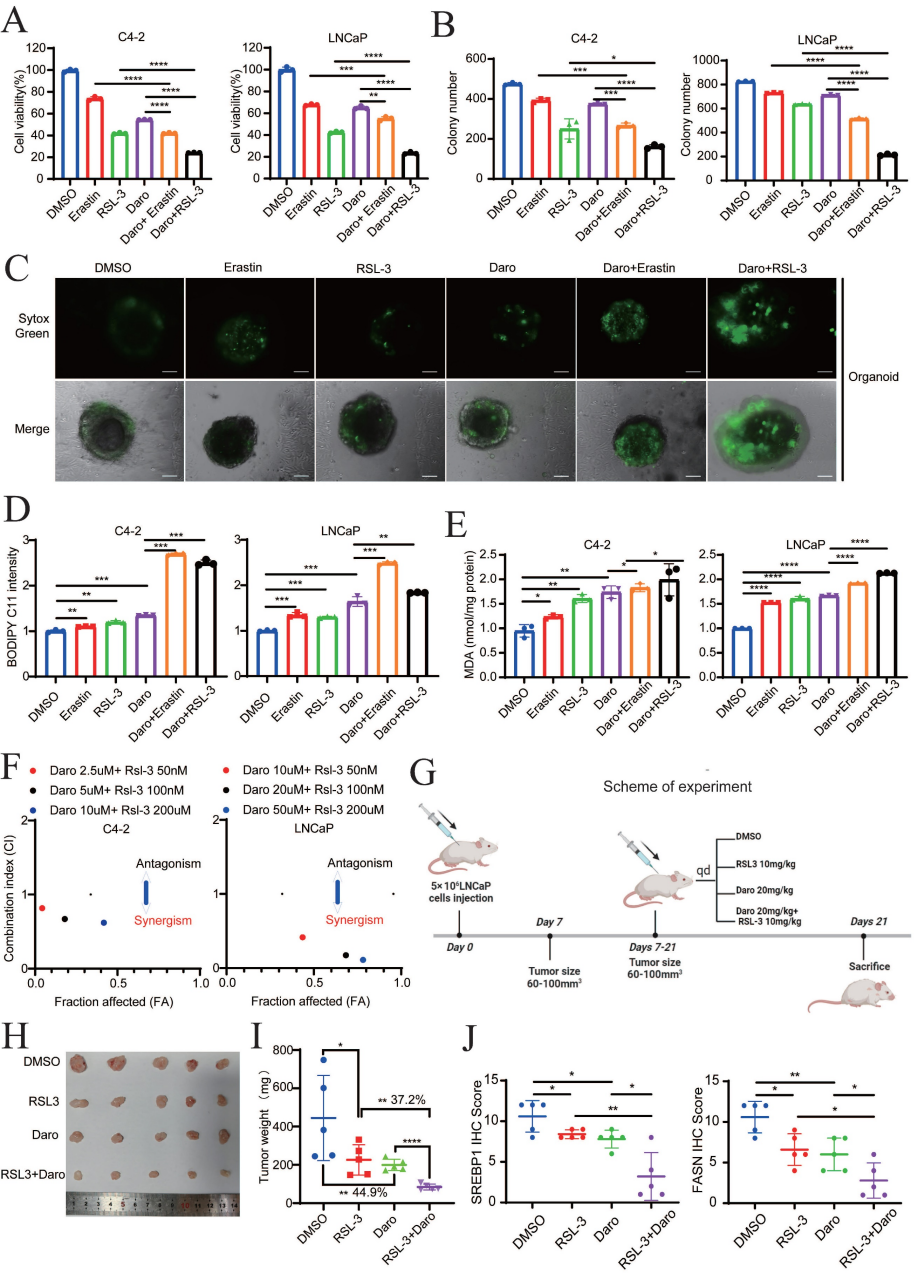
** Darolutamide and FINs synergistically sensitize PCa cells to cell death. A** Viability analysis of C4-2 and LNCaP cells treated with darolutamide (C4-2,50 µM; LNCaP, 10 µM) or FINs (erastin, 20 µM and RSL-3,100 nM) or their combination for 24 h. **B** Quantification analysis of colony formation in C4-2 or LNCaP cells with indicated treatments.** C** Representative images of SYTOX Green assays in prostate cancer organoid models subjected to the indicated treatments. **D-E** Lipid peroxidation levels (**D**) and MDA concentrations (**E**) examined in C4-2 and LNCaP cells treated with indicated treatments. **F** The synergistic effect of the combination of darolutamide and RSL-3 on antitumor activity using CalcuSyn software. **G** Treatment schema for nude mice bearing LNCaP xenografts. **H** Representative images of xenograft tumors in each group treated with darolutamide and/or RSL-3 at the experimental endpoints. **I** Tumor weights in each group treated with darolutamide and/or RSL-3. **J** Quantitative analysis of SREBP1 and FASN expression in tumor sections from mice given the indicated treatments. *p < 0.05, **p < 0.01, and ***p < 0.001.

To further explore the role of darolutamide in ferroptosis, the effect of the combination of darolutamide and ferroptosis inducers (FINs), such as erastin or RSL3, on cell death was examined. Cell viability assays of both C4-2 and LNCaP cells treated with darolutamide and/or FINs at the indicated concentrations revealed that combination treatment significantly reduced cell viability (**Fig. 8A**). Compared to treatment with the single drugs, darolutamide combined with FINs significantly inhibited clonogenic survival in C4-2 and LNCaP cells (**Fig. 8B, Fig. S8A**). In addition, SYTOX Green assays using a PCa organoid model showed that cell death was significantly increased after the combination treatment (**Fig. 8C**). BODIPY™ 581/591C11 staining and MDA assays showed that FINs not only markedly triggered lipid peroxidation, but also synergistically potentiated darolutamide-induced lipid peroxidation in C4-2 and LNCaP cells (**Fig. 8D-E**). To test the synergistic effect, PCa cells were treated with various concentrations of darolutamide, erastin, or darolutamide in combination with erastin. CompuSyn software analysis based on the Chou-Talalay methodology showed that the combination indices at the indicated concentrations of erastin or RSL-3 and darolutamide were less than 1 in both C4-2 and LNCaP cells (**Fig. 8F, Fig. S8B**), suggesting that FINs synergize with darolutamide. Taken together, these data clearly indicated that FINs and darolutamide synergistically sensitize PCa cells to death *in vitro*.

To further confirm the synergistic efficacy of darolutamide and FINs *in vivo*, LNCaP cells were injected into the right flank of 4-week-old male BALB/c nude mice (**Fig. 8G**). Compared to the control group, the RSL-3 or darolutamide group exhibited decreased tumor size and tumor growth, whereas the combined treatment group achieved the greatest suppression of tumor growth (**Fig. 8H-I, Fig. S8C**). The weights of the mice in these groups did not differ, indicating the drug safety of darolutamide and RSL-3* in vivo* (**Fig. S8D**). In the combination treatment group, the expression levels of SREBP1, FASN, and Ki-67 were significantly decreased, while the expression level of 4-HNE, a marker of ferroptosis, was significantly increased (**Fig. 8J, Fig. S8E-F**). In addition, 4-HNE expression was negatively correlated with the expression of SREBP1 and FASN. As expected, the expression levels of SREBP1 and FASN expression were significantly positively correlated (**Fig. S8G**). Collectively, these results showed that darolutamide and FINs synergistically sensitize PCa cells to cell death both *in vitro* and *in vivo*.

## Discussion

**Figure 9:**
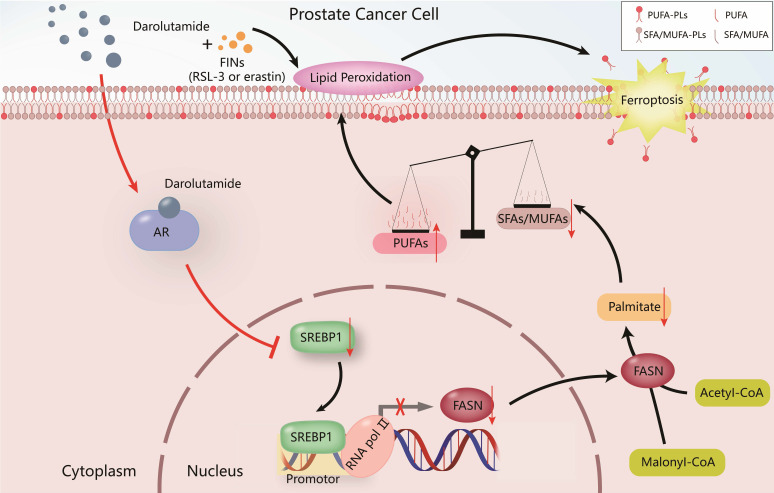
Proposed model of the mechanism by which darolutamide regulates ferroptosis through the SREBP1-FASN axis in PC.

PCa is the most common malignant tumor in males according to the cancer statistics in the United States 32, and it greatly increases the economic burden worldwide and reduces the lifespan of patients. Hormone therapy can initially reduce the PSA level and improve the overall survival rate in advanced PCa patients, but almost all patients ultimately develop therapy resistance, such as CRPC patients. Various studies have shown that one of the most important reasons for CRPC progression is the dysregulation of the AR variant 3, 6. Darolutamide, especially antagonist of mutated AR, is approved by the FDA for the treatment of CRPC patients who are resistant to apalutamide or enzalutamide 6. The present study demonstrated that darolutamide significantly induced ferroptosis in PCa cells, especially in the presence of AR splice variants. Mechanistically, darolutamide facilitated ferroptosis through regulating the imbalance between PUFAs and SFAs via the SREBP1-FASN axis. Darolutamide combined with FINs significantly inhibited PCa tumorigenesis, indicating that the combination treatment is a promising and effective strategy for treating PCa (**Fig. 9**).

Lipid metabolism is a vital process for PCa tumorigenesis and progression, and targeting key genes involved in lipid metabolism regulation may inhibit PCa development. Ferroptosis, a new form of cell death, is characterized by iron-dependent lipid peroxidation. Recently, several studies have demonstrated that there is a close intersection between lipid metabolism and ferroptosis. For example, mutant KRAS in lung cancer can be significantly inhibited by targeting the land cycle to promote the accumulation of lipid metabolism prone to intracellular oxidation 33. Additionally, the lipidomic analysis has demonstrated that oleic acid inhibits the accumulation of ROS, thus preventing ferroptosis in breast cancer 34. However, it remains unknown whether there is a relationship between darolutamide and ferroptosis. A previous study has shown that AR variants confer resistance to ferroptosis induced by antiandrogen drugs, which may drive castration resistance in PCa 35. The present study revealed that ferroptosis and oxidative phosphorylation are involved in AR mutated PCa. However, it remains unclear whether darolutamide participates in lipid peroxidation. The present study demonstrated that darolutamide mediates ferroptosis onset by downregulating SREBP1 expression.

SREBP1, the main regulatory element of lipid metabolism, has recently been related to poor clinical prognosis in different cancers, and it is involved in ferroptosis onset 36. Research has shown that the mTOR-SREBP1 axis suppresses the ferroptosis activity via the accumulation of MUFAs 18, 37. Moreover, second-generation androgen receptor antagonists participate in the PI3K-AKT-mTOR signaling pathway 38, suggesting that there is underlying crosstalk between ARI and ferroptosis. Previous studies have focused mainly on the SREBP1-SCD1 axis, especially on SCD1-mediated lipogenesis. Ectopic overexpression of SREBP1 or SCD1 regulates the sensitivity to ferroptosis induction 17. The present study demonstrated that SREBP1 regulates ferroptosis through mediating FASN transcription by directly binding to the FASN promoter and recruiting RNA polymerase II instead of modulating SCD1 expression, indicating that the SREBP1-FASN axis may be more important in mediating ferroptosis in PCa.

FASN is the core downstream gene of SREBP1 and FASN inhibition enhances susceptibility to lipid oxidative stress, leading to ferroptosis in acute myeloid leukemia and lung cancer 33, 39. However, it remains unknown whether FASN mediates ferroptosis in PCa. The present study demonstrated that SREBP1 directly binds to the FASN promoter and recruits RNA polymerase II to activate FASN transcription. Moreover, BODIPY C11 and MDA analysis demonstrated that FASN overexpression significantly reverses the effect of AR knockdown on ferroptosis, indicating that FASN participates in AR-induced ferroptosis. PCa is characterized by aberrant lipid metabolism, and targeting lipid metabolism-related genes can inhibit PCa progression by decreasing lipid synthesis 40, 41. For example, 2,4-dienoyl CoA reductase 2 (DECR2) knockdown significantly influences lipid composition, especially increasing the abundance of MUFAs and PUFAs, as well as increasing the sensitivity to enzalutamide 41. FASN, also a central regulator of de novo lipogenesis, plays a pivotal role in tumor growth and serves as a fuel source for ATP generation. Accumulating evidence has shown that FASN is overexpressed in cancer and promotes cancer progression 42, 43. However, the relationship between FASN and PCa is still unclear. The present results showed that FASN expression is significantly higher in PCa than in all other types of cancer in TCGA database, suggesting the unique and critical role of FASN in PCa. Moreover, FASN expression significantly increases with advanced clinicopathological tumor stage, increased Gleason score, and poor DFS, further indicating that FASN plays a vital role in PCa tumorigenesis and progression. Thus, FASN may be a promising new and attractive therapeutic target for PCa treatment.

Lipidomic analysis suggested that FASN knockdown increases PE/PC-PUFAs, while decreases PE/PC-MUFAs and PE/PC-SFAs, leading to an imbalance and ferroptosis induction. Palmitate acid is the most abundant saturated fatty acid in humans and is synthesized by FASN catalysis. SFAs and MUFAs can modify the ratio of membrane lipids, leading to decreased lipid peroxidation. The present study demonstrated that palmitate, the downstream product of FASN, rescues the effect of FASN knockdown and restores resistance to ferroptosis, indicating that FASN knockdown-mediated ferroptosis mainly depends on palmitate. Further investigations of other MUFAs and SFAs are needed to explore the underlying mechanism of ferroptosis regulation and potential clinical applications for PCa treatment.

The pharmacologic effects of traditional AR inhibitors (enzalutamide, bicalutamide, and apalutamide) are based on the inhibition of androgen binding to the ligand binding domain (LBD), leading to the suppression of AR nuclear translocation and the transcription of AR target genes, such as PSA or FKBP51. These AR inhibitors represent breakthroughs and improve the survival time of PCa patients; however, 20 to 40% of patients acquire secondary resistance due to AR splice variants 3, 5. Because AR variants still have the ability to bind DNA in the absence of androgen, patients with AR variants confer resistance to AR inhibitors. Moreover, AR-V7 has been found to be related to shorter survival time and worse prognosis in PCa patients. Therefore, targeting AR-V7 for therapeutic selection in particular PCa patients is urgently needed. Darolutamide is a unique AR inhibitor that antagonizes AR mutants and has a low brain distribution, thus prolonging survival time of PCa patients, especially nonmetastatic CRPC patients 5. The present study demonstrated that darolutamide significantly increases the efficacy of FINs against ferroptosis both *in vivo* and *in vitro*, indicating that this combination is a potential synergistic treatment for PCa, especially for patients with AR variants. In addition, ferroptosis enhances tumor immunogenicity and crosstalk with immune cells, especially T cells or macrophages 44. Therefore, the efficacy and safety of triple therapy combining darolutamide, FINs, and immunotherapy, for the treatment of PCa deserve further exploration.

## Conclusions

Taken together, the present findings showed that darolutamide promotes ferroptosis via inhibiting the SREBP1-FASN axis in PCa. Targeting SREBP1-FASN interrupts the balance between PUFAs and SFAs, thereby inducing lipid peroxidation onset in PCa. The present study suggested a potential strategy for combining darolutamide with FINs for PCa treatment.

## Supplementary Material

Supplementary figures and information.

Supplementary data.
