# Supplementary material for: Darolutamide-mediated phospholipid remodeling induces ferroptosis through the SREBP1-FASN axis in prostate cancer
Source: Int J Biol Sci. 2024 Sep 3;20(12):4635–53. doi: 10.7150/ijbs.101039 (PMC11414384; doi:10.7150/ijbs.101039)
Supplement: Supplementary file 1 — Supplementary figures and information. [file ijbsv20p4635s1.pdf]

Supplementary file

1 shRNA, qRT-PCR primer and ChIP assay primer sequences

shCtrl targeting sequence:TTCTCCGAACGTGTCACGT

shAR#1 targeting sequence: GCAGAAATGATTGCACTAT

shAR#2 targeting sequence: GCACCTCTCTCAAGAGTTT

shSREBP1#1 targeting sequence: GGAGCCATGGATTGCACTT

shSREBP1#2 targeting sequence: GCCATCGACTACATTCGCT

shFASN#1 targeting sequence: GCACCAATACAGATGGCTT

shFASN#2 targeting sequence: GGGACAGTGCATCAAAGAA

GAPDH qRT-PCR Forward primer: CAAGGCTGAGAACGGGAAG

GAPDH qRT-PCR Reverse primer: TGAAGACGCCAGTGGACTC

SREBP1 qRT-PCR Forward primer: ACTTCTGGAGGCATCGCAAGCA

SREBP1 qRT-PCR Reverse primer: AGGTTCCAGAGGAGGCTACAAG

ACC qRT-PCR Forward primer: TTCACTCCACCTTGTCAGCGGA

ACC qRT-PCR Reverse primer: GTCAGAGAAGCAGCCCATCACT

ACLY qRT-PCR Forward primer: GCTCTGCCTATGACAGCACCAT

ACLY qRT-PCR Reverse primer: GTCCGATGATGGTCACTCCCTT

FASN qRT-PCR Forward primer: TTCTACGGCTCCACGCTCTTCC

FASN qRT-PCR Reverse primer: GAAGAGTCTTCGTCAGCCAGGA

SCD1 qRT-PCR Forward primer: CCTGGTTTCACTTGGAGCTGTG

SCD1 qRT-PCR Reverse primer: TGTGGTGAAGTTGATGTGCCAGC

AR qRT-PCR Forward primer: ATGGTGAGCAGAGTGCCCTATC

AR qRT-PCR Reverse primer: ATGGTCCCTGGCAGTCTCCAAA

PSA qRT-PCR Forward primer: CGCAAGTTCACCCTCAGAAGGT

PSA qRT-PCR Reverse primer: GACGTGATACCTTGAAGCACACC

FKBP51 qRT-PCR Forward primer: GCGAAGGAGAAGACCACGACAT

FKBP51 qRT-PCR Reverse primer: TAGGCTTCCCTGCCTCTCCAAA

AR qRT-PCR Forward primer: ATGGTGAGCAGAGTGCCCTATC

AR qRT-PCR Reverse primer: ATGGTCCCTGGCAGTCTCCAAA

FASN ChIP Forward primer:CTTCCCTTGTCCTTCCTTGACC

FASN ChIP Reverse primer: TTGTGAGGCCACAGGTGTCT

Supplementary Figure Legends

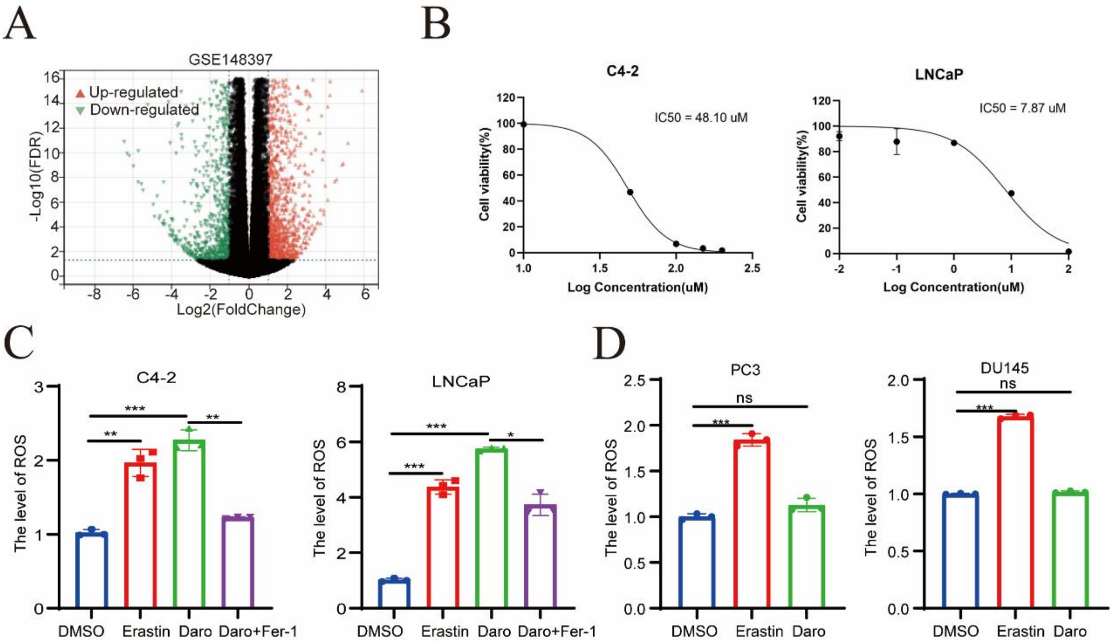

Supplementary Fig.1 Darolutamide promotes ferroptosis in AR+ PCa cells. A Volcano plots of DEGs in darolutamide-treated AR+ prostate cancer cells (LOG2|FC| ≥ 1, and p ≤ 0.05). **B** IC50 of darolutamide in C4-2 and LNCaP cells. **C** ROS levels in C4-2 and LNCaP cells treated with darolutamide in the presence or absence of Fer-1 using ROS assay kit. **D** ROS levels in PC3 and DU145 cells treated with erastin (20μM) or darolutamide (50μM) using ROS assay kit. *p < 0.05, **p < 0.01, and ***p < 0.001.

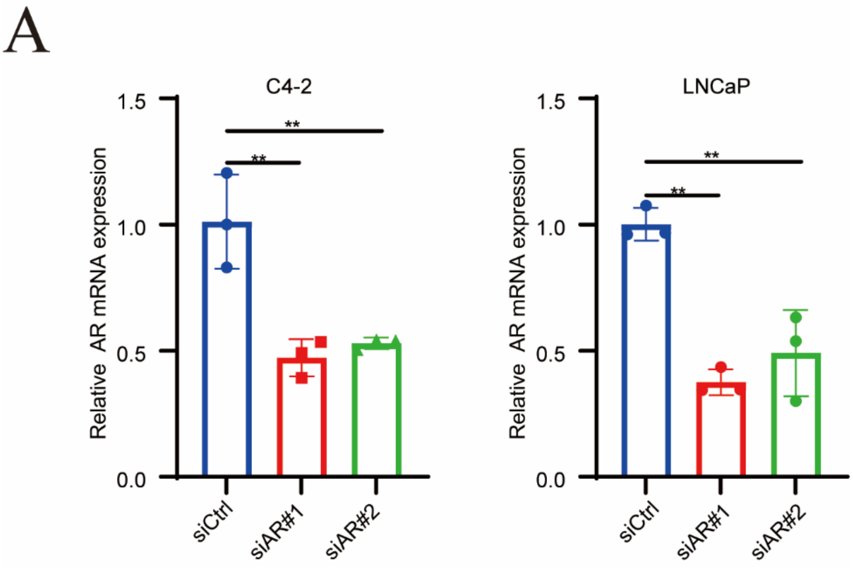

Supplementary Fig.2 AR deletion sensitizes PCa cells to ferroptosis. A Relative mRNA level of AR in indicated cells treated with siCtrl and siRNA targeting AR. *p < 0.05, **p < 0.01, and ***p < 0.001.

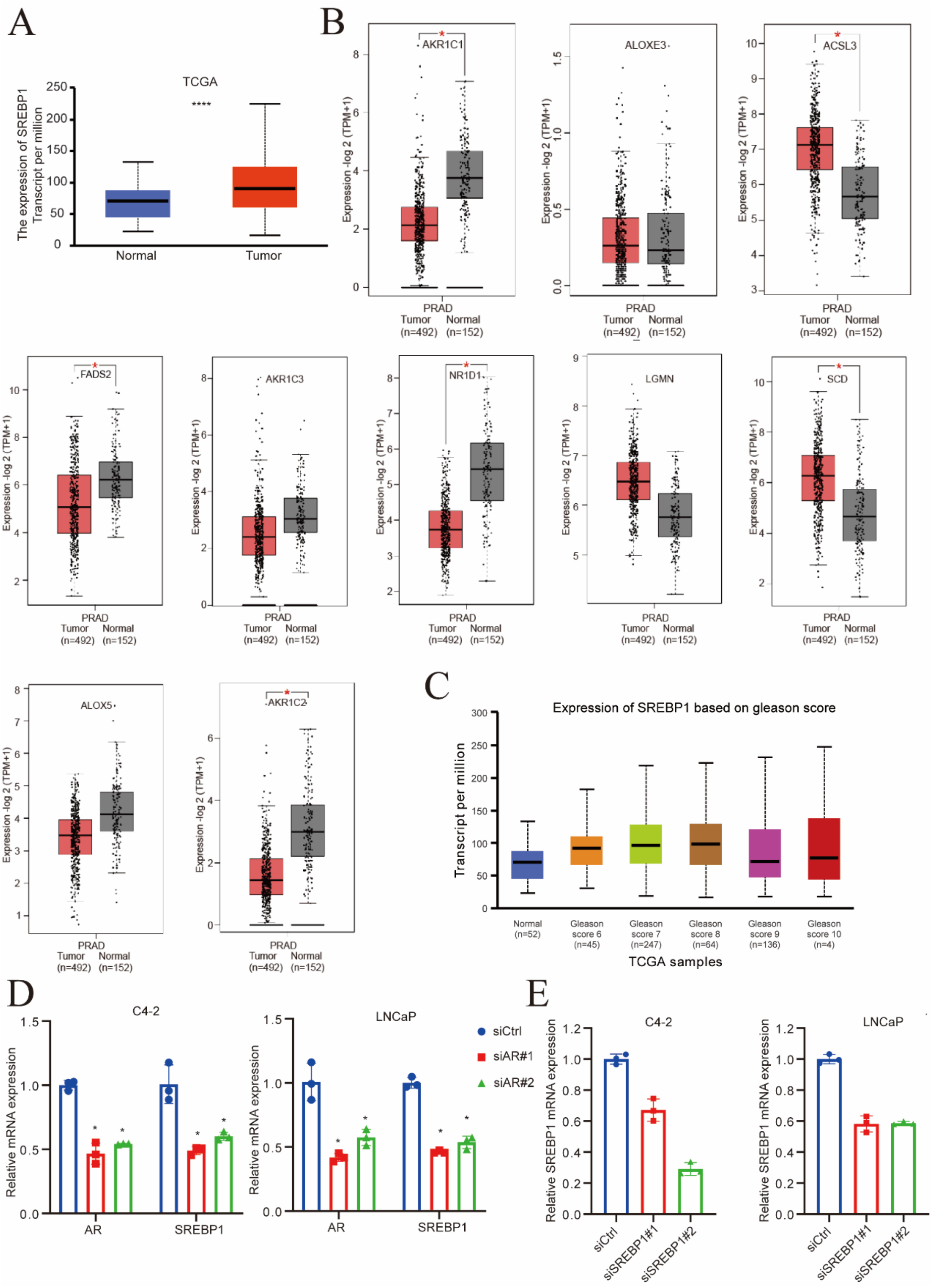

Supplementary Fig.3 Darolutamide promotes ferroptosis by inhibiting SREBP1 expression. A-B Analysis of indicated genes expression between PCa cancer and normal samples in TCGA database. **C** Gleason score of SREBP1 in TCGA database. **D-E** Relative mRNA level of AR and SREBP1 in indicated cells treated with siCtrl and siRAN targeting AR or SREBP1. $*p < 0.05$, $**p < 0.01$, and $***p < 0.001$.

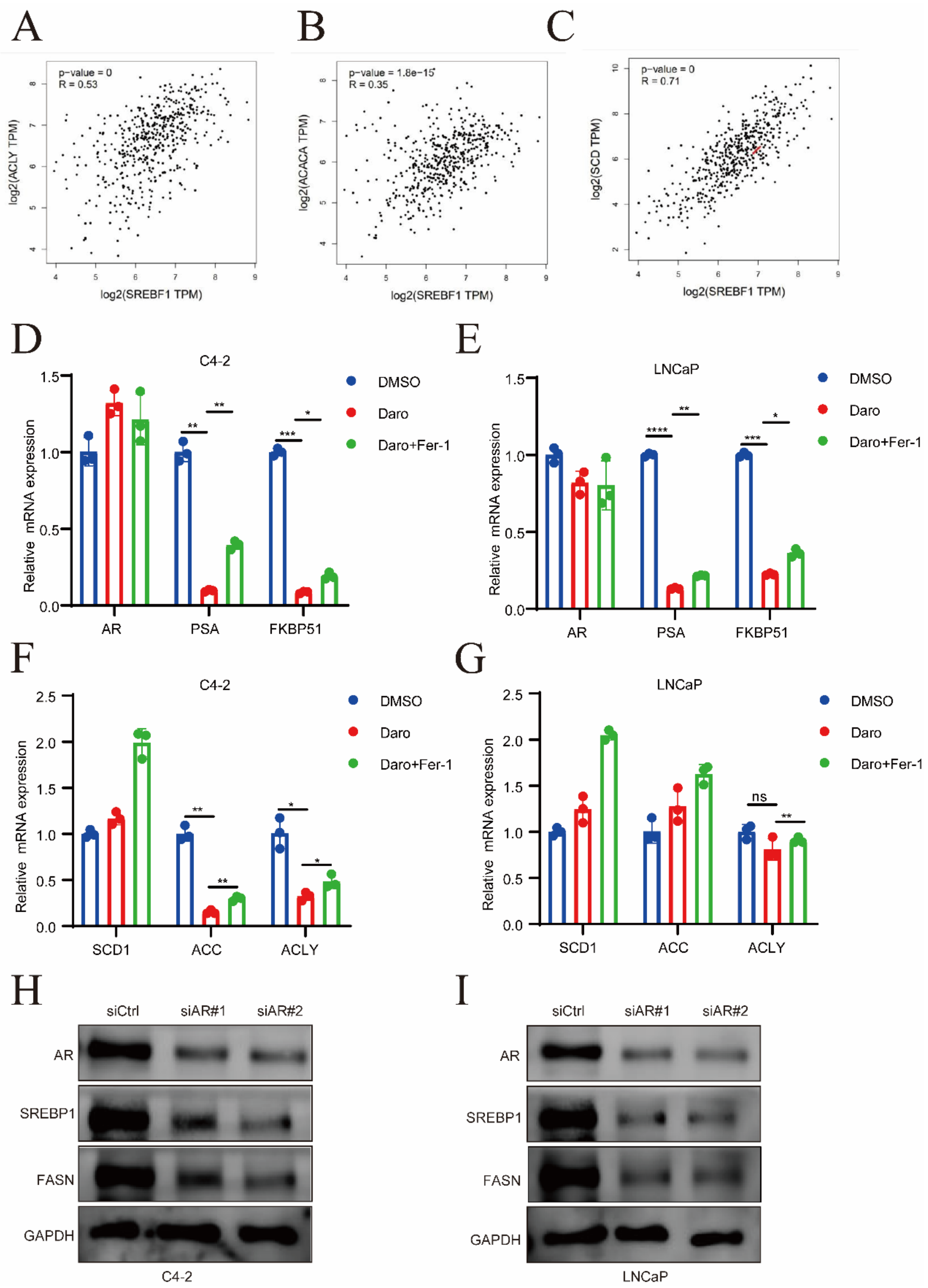

Supplementary Fig.4 Darolutamide facilitates ferroptosis by regulating the SREBP1-FASN axis in PCa. A-C The correlation analysis between SREBP1 and targeted genes (ACC, ACLY, and SCD1) in TCGA database. **D-E** Relative mRNA level of AR and downstream genes (PSA and FKBP51) in indicated cells treated with darolutamide or combined with Fer-1. **F-G** Relative mRNA level of the SREBP1-related targeted genes (ACC, ACLY, and SCD1) in indicated cells treated with darolutamide or combined with Fer-1. **H-I** Immunoblot analysis of AR, SREBP1,

and FASN expression in indicated cells with AR knockdown. *$p < 0.05$, **$p < 0.01$, and ***$p < 0.001$.

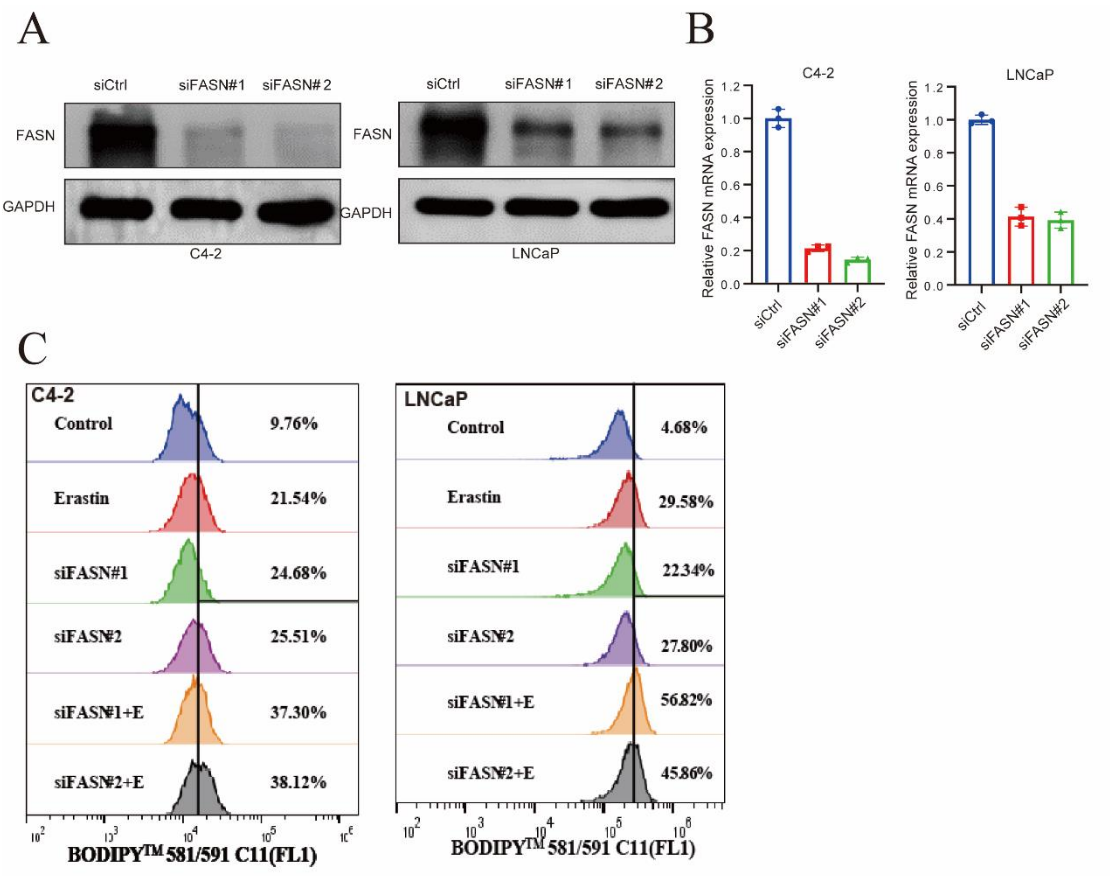

Supplementary Fig.5 FASN inhibition induces lipid peroxidation in PCa cells. A Immunoblot analysis of FASN expression in FASN-deficient C4-2 and LNCaP cells. **B** Relative mRNA level of FASN in indicated cells with FASN knockdown. **C** Levels of BODIPY C11, as measure of the lipid peroxidation, examined in indicated groups with or without erastin for 24h using FlowJo software.. *$p < 0.05$, **$p < 0.01$, and ***$p < 0.001$.

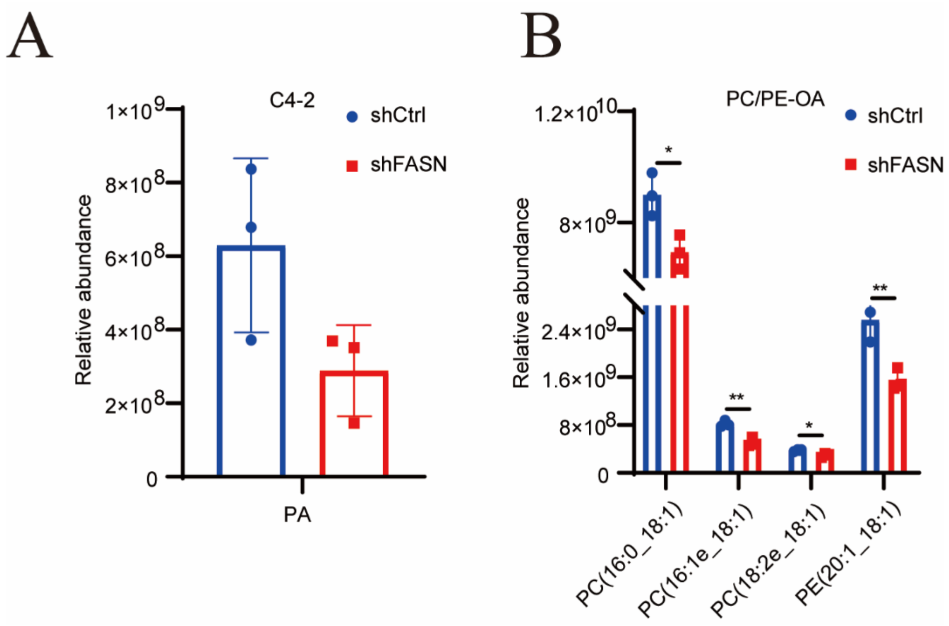

Supplementary Fig.6 FASN inhibition mediates the SFA/PUFA ratio to regulate ferroptosis in PCa. A Quantification of PA in shCtrl and shFASN cells, as indicated. **B** Quantification of PC/PE-OA in LNCaP with shCtrl or shFASN knockdown groups. $*p < 0.05$, $**p < 0.01$, and $***p < 0.001$.

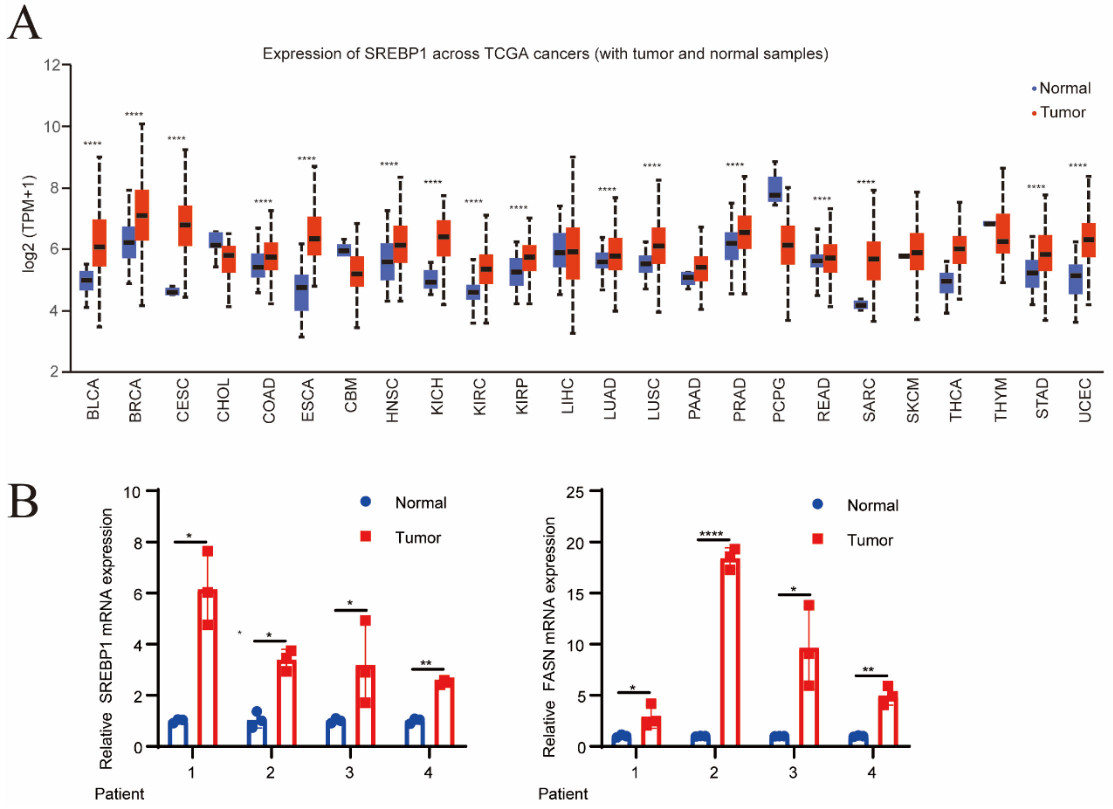

Supplementary Fig.7 SREBP1 and FASN are elevated in PCa and are associated with poor prognosis. A Analysis of SREBP1 expression in pan-cancer using The Cancer Genome Atlas datasets. **B** Relative mRNA level of SREBP1 in SYSMH cohort. $*p < 0.05$, $**p < 0.01$, and $***p < 0.001$.

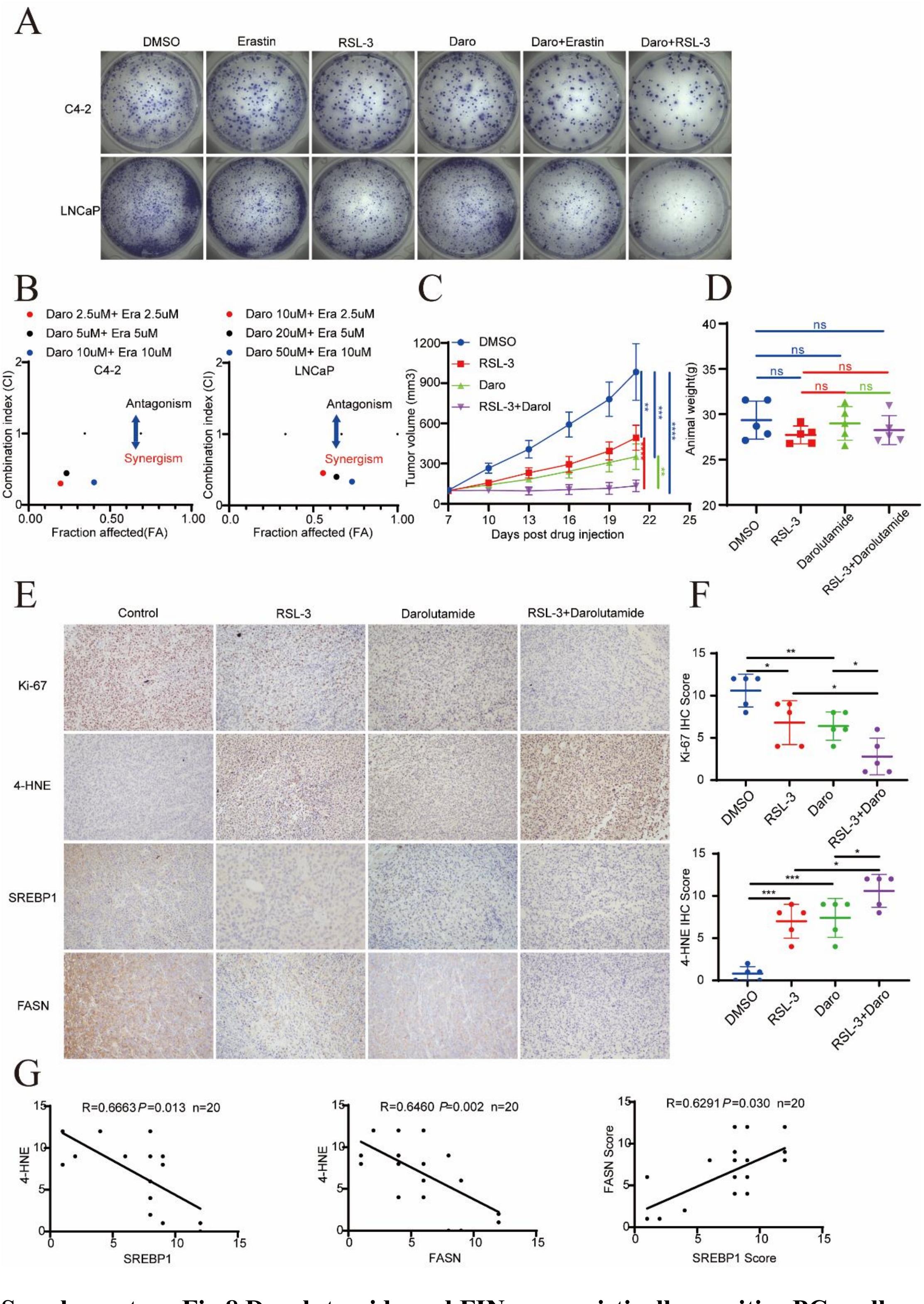

Supplementary Fig.8 Darolutamide and FINs synergistically sensitize PCa cells to cell death. A Representative images of colony formation analysis of C4-2 or LNCaP cells treated with indicated groups. **B** The Chou-Talalay plot showing the combination effect of darolutamide and erastin treatment. **C** The tumor volume in each group treated with darolutamide and/or RSL-3. **D** The animal weight in each group treated with darolutamide and/or RSL-3. **E** Representative immunohistochemistry images of Ki67, 4-HNE, SREBP1, and FASN in the indicated

xenograft tumors. **F** Quantitative analysis of Ki67 and 4-HNE expression in indicated treatment. **G** Correlation analysis between SREBP1 and FASN, SREBP1 and 4-HNE, FASN, and 4-HNE expression using the Pearson correlation coefficient test, respectively. *$p < 0.05$, **$p < 0.01$, and ***$p < 0.001$.
